# CILP2: From ECM Component to a Pleiotropic Modulator in Metabolic Dysfunction, Cancer, and Beyond

**DOI:** 10.3390/biom16010167

**Published:** 2026-01-19

**Authors:** Zheqiong Tan, Suotian Liu, Zhongxin Lu

**Affiliations:** Department of Medical Laboratory, The Central Hospital of Wuhan, Tongji Medical College, Huazhong University of Science and Technology, Wuhan 430014, China; 2021110549@stu.cqmu.edu.cn

**Keywords:** CILP2, extracellular matrix, metabolic syndrome, cancer, biomarker

## Abstract

Initially characterized as a component of the extracellular matrix (ECM) in cartilage, cartilage intermediate layer protein 2 (CILP2) is now recognized as a pleiotropic secretory protein with far-reaching roles in physiology and disease. This review synthesizes evidence establishing CILP2 as a key modulator at the nexus of metabolic dysfunction, cancer, and other pathologies. Genomic studies have firmly established the NCAN-CILP2 locus as a hotspot for genetic variants influencing dyslipidemia and cardiovascular risk. Functionally, CILP2 is upregulated by metabolic stress, including high glucose and oxidatively modified LDL (oxLDL), and actively contributes to pathologies such as dyslipidemia, diabetes, and sarcopenia by impairing glucose metabolism and mitochondrial function. Its role extends to fibrosis and neurodevelopment, promoting hypertrophic scar formation and neurogenesis through interactions with ATP citrate lyase (ACLY) and Wnt3a, respectively. More recently, CILP2 has emerged as an oncoprotein, overexpressed in multiple cancers, including pancreatic ductal adenocarcinoma and colorectal cancer. It drives tumor proliferation and metastasis and correlates with tumor microenvironment remodeling through mechanisms involving Akt/EMT signaling and immune infiltration. The dysregulation of CILP2 in patient serum and its correlation with disease severity and poor prognosis highlight it as a promising biomarker and a compelling therapeutic target across a spectrum of human diseases.

## 1. Introduction

The extracellular matrix (ECM) is not merely a static scaffold for cellular attachment but also a dynamic and bioactive entity that critically regulates tissue homeostasis, cell signaling, and disease pathogenesis [1]. In cancer, the ECM undergoes profound remodeling, becoming dysregulated in its density and composition, which in turn shapes a tumor-permissive microenvironment that fosters growth, invasion, and therapeutic resistance [2]. This has spurred growing interest in targeting the ECM for novel therapeutic strategies.

Among the myriad components of the ECM, the cartilage intermediate layer proteins (CILPs) have emerged as molecules of increasing significance. The CILP family comprises two highly homologous secretory glycoproteins: CILP1 and CILP2 [3]. CILP1 was first identified in 1998 and named for its deposition in the intermediate zone of articular cartilage [4]. CILP2, a homologue to CILP1, was first discovered in mouse cartilage by means of transcriptomics. The cDNAs of CILP1 and CILP2 are 51.5% identical, and the proteins of CILP1 and CILP2 are 66% conserved on an amino acid basis [5]. Compared to CILP1, CILP2 appears to be more abundant in the deeper middle region of the articular cartilage, associated with the maintenance of joint stability [6]. However, its expression is not confined to cartilage; it is also expressed in muscle, heart, brain, skin, and tumor tissues, hinting at broader physiological and pathological functions [7]. As a secretory protein in the ECM, CILP2 is detected in human serum [8,9,10]. It is not merely a passive structural element but also a dynamic, responsive player upregulated by metabolic stressors like high glucose, oxidized lipids, and oxidative stress, which actively contributes to pathologies including diabetes, dyslipidemia, atherosclerosis, and sarcopenia. In addition, genome-wide association studies (GWAS) have pinpointed the NCAN-CILP2 locus on chromosome 19p13 as a hotspot for genetic variants influencing plasma lipid levels and the risk of metabolic diseases [11,12,13].

More recently, the purview of CILP2 has expanded into oncology. It is overexpressed in a wide array of cancers and correlates with poor prognosis of patients. It promotes tumor proliferation and metastasis in pancreatic ductal adenocarcinoma (PDAC) and colorectal cancer (CRC) through the regulation of epithelial–mesenchymal transition (EMT) or tumor microenvironment (TME) remodeling [7,14,15].

This review aims to synthesize the accumulating evidence that positions CILP2 at the nexus of multiple pathological processes. We will trace its evolution from an ECM constituent to a multifunctional signaling modulator, delving into its intricate roles in the regulation of metabolic reprogramming, neurodevelopment, EMT modulation, and TME remodeling. By integrating insights from genetic, molecular, and clinical studies, we seek to illuminate the pathogenic mechanisms of CILP2 and evaluate its burgeoning potential as a biomarker and therapeutic target across a spectrum of human diseases.

## 2. Structure of CILP2

**Figure 1:**
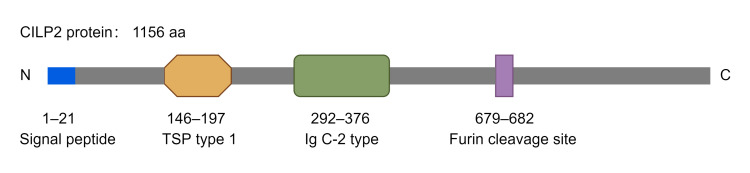
Domain structure of CILP2 protein. CILP2 protein is composed of a polypeptide of 1,156 amino acids, which can be cleaved into free N-terminal and C-terminal domains at a furin endoprotease consensus site. The N-terminal domain of CILP2 comprises a putative signal peptide of 21 amino acids, a conserved TSP type I domain, and an Ig C-2 type domain.

The human CILP2 gene resides on chromosome 19p13, encoding a polypeptide of 1156 amino acids with a molecular mass of 126.3 kDa [6]. CILP2 can be cleaved into distinct, free N-terminal and C-terminal domains at a furin endoprotease consensus site (Figure 1). Apart from a putative signal peptide of 21 amino acids, the N-terminal domain of CILP2 has a substantially conserved thrombospondin (TSP) type I repeat domain and an immunoglobulin (Ig) C-2 type domain, which are conserved in CILP1 [5]. However, CILP2 does not share the putative motifs for the aldehyde dehydrogenase and ATP-binding in the N-terminal domain of CILP1, leading to the lack of insulin-like growth factor-1 (IGF-1) antagonism in CILP2 [16,17]. The C-terminal domain of CILP2 was highly conserved in CILP1, corresponding to a homologue of porcine nucleotide pyrophosphatase phosphodiesterase (NPP), but was later identified as having no nucleotide NPP activity [5].

Structurally, the presence of a thrombospondin-1 (TSP-1) domain in CILP2 provides critical insights into its potential regulatory interactions and biological functions. The three type 1 repeated sequence motifs in the TSP-1 domain are reported to function as protein- and glycosaminoglycan (GAG)-binding sites in other proteins, which facilitate substrate recognition and the interaction with extracellular or membrane proteins. The W(S/G)XW and CSVTCG motifs within the TSP-1 domain are able to bind thrombospondin and CD36, respectively. Moreover, the W(S/G)XW motif in CILP1 binds to transforming growth factor beta 1 (TGF-β1) and inhibits the TGF-β1/SMAD signaling pathway [3]. Given that the TSP-1 domain is conserved in CILP2, CILP-2 may have the potential to recognize and bind to the above or other ECM components and growth factors, which requires direct experimental validation through mass spectrometry and protein-protein binding assays.

## 3. The Functions and Mechanisms of CILP2 in Diseases

**Figure 2:**
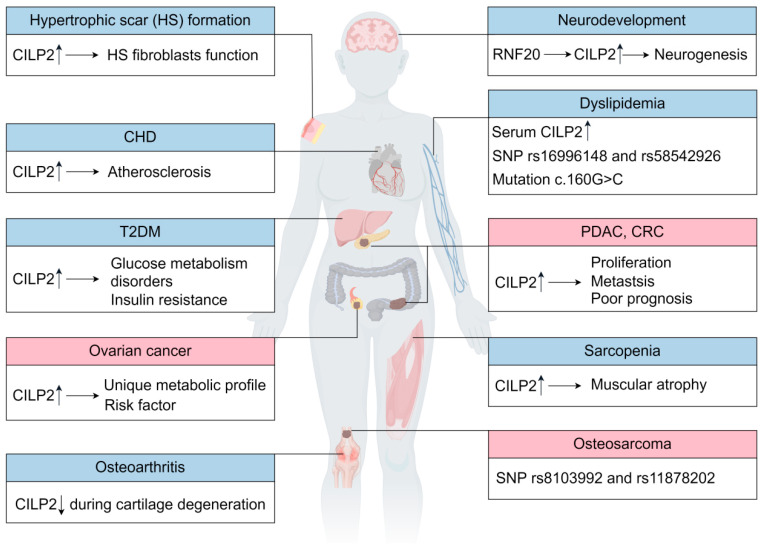
The roles of CILP2 in human diseases. CILP2 promotes the progression of T2DM, CHD, sarcopenia, HS formation, PDAC, CRC, and ovarian cancer. In contrast, CILP2 supports joint stability and neurodevelopment. Additionally, the SNPs rs16996148 and rs58542926 are associated with dyslipidemia, whereas the height-associated SNPs rs8103992 and rs11878202 are linked to osteosarcoma risk. The red and blue colors correspond to dysregulation of CILP2 in cancer and in other diseases, respectively, as shown.

Far beyond its initial structural role in cartilage, CILP2 has emerged as a key pathogenic driver in a broad spectrum of diseases. This section systematically delineates the pleiotropic functions and underlying mechanisms of CILP2, spanning osteoarthritis, metabolic disorders, fibrosis, neurodevelopment, and oncogenesis (Table 1, Figure 2). Evidence will be presented for how CILP2 engages specific pathways and molecular interactions to disrupt tissue homeostasis and fuel disease progression. The diverse functions of CILP2 in the regulation of ECM modification, metabolic reprogramming, neurodevelopmental, EMT, and TME remodeling reflect its pleiotropic nature and highlight its potential as a cross-disease biomarker and therapeutic target.

### 3.1. ECM Organization and Remodeling

CILP2 plays a fundamental role in ECM assembly and structural integrity, partic-ularly in cartilage. During the development of a mouse knee joint, CILP2 was initially confined to the surface of the mouse articular cartilage, becoming widely expressed in the deeper intermediate zone of the articular cartilage at maturity [6]. Notably, CILP2 expression was reduced in surgically induced osteoarthritis in mice, whereas CILP1 expression was increased in articular cartilage, highlighting CILP2’s role as a potential biomarker of cartilage damage [6]. Most CILP2 protein in mouse articular cartilage is proteolytically processed and cleaved at the furin cleavage site, which likely facilitates its integration into the ECM network [6]. Furthermore, CILP2 was visualized to be located in close association with large cross-banded fibrils and collagen VI particles detected via immunogold electron microscopy, suggesting a potential interaction between CILP2 and collagen VI, or larger multimeric suprastructures formed by CILP2 and collagen VI-containing microfibrils in human articular cartilage.

Beyond cartilage, CILP2 actively drives pathological ECM remodeling in fibrosis. In the process of normal wound repair, fibroblasts can be activated and differentiate into myofibroblasts, which facilitate wound contraction by migrating to the damaged area and producing ECM components [30]. Abnormal wound repair processes will result in the formation of a hypertrophic scar (HS), characterized by the abnormal activation and proliferation of myofibroblasts and excessive deposition of ECM [31].

CILP2 was elevated in the serum and HS tissues of human HS patients compared to healthy individuals in a clinical cohort, indicating that CILP2 could be a potential biomarker for HS [10]. Mouse and rabbit HS models also demonstrated that CILP2 was significantly overexpressed in HS tissues and colocalized with α-SMA in skin fibroblasts [10]. Mechanistically, CILP2 was found to interact with ACLY, a crucial enzyme that generates acetyl-CoA for protein acetylation and fatty acid synthesis. CILP2 stabilized ACLY protein by blocking the ubiquitination degradation of ACLY, which promoted the acetylation-dependent stabilization of Snail, a vital transcription factor for tissue fibrosis development [10,32,33]. Silencing CILP2 suppressed the activation, proliferation, migration, collagen contraction capacity, and expression of fibrosis-associated molecules in hypertrophic scar fibroblasts (HSFs), while attenuating hypertrophic scar formation in mice by reducing dermis/epidermis thickness, collagen deposition/arrangement, and expression of fibrosis markers (α-SMA, COL I, and COL III), underscoring its central role in fibrotic tissue remodeling [10].

Importantly, CILP2’s influence extends to cancer-associated ECM dysregulation. In colorectal cancer (CRC), CILP2 was overexpressed in tumor tissues [14,34]. Gene function, gene enrichment, and KEGG analyses indicated that CILP2 expression was closely related to ECM–receptor interaction and the TGF-β signaling pathway, which are critical for tumor stroma remodeling and metastatic progression [15]. Silencing CILP2 reduced the expression of ECM degradation proteins—matrix metalloproteinase 2 (MMP2) and MMP9 [14].

**Figure 3:**
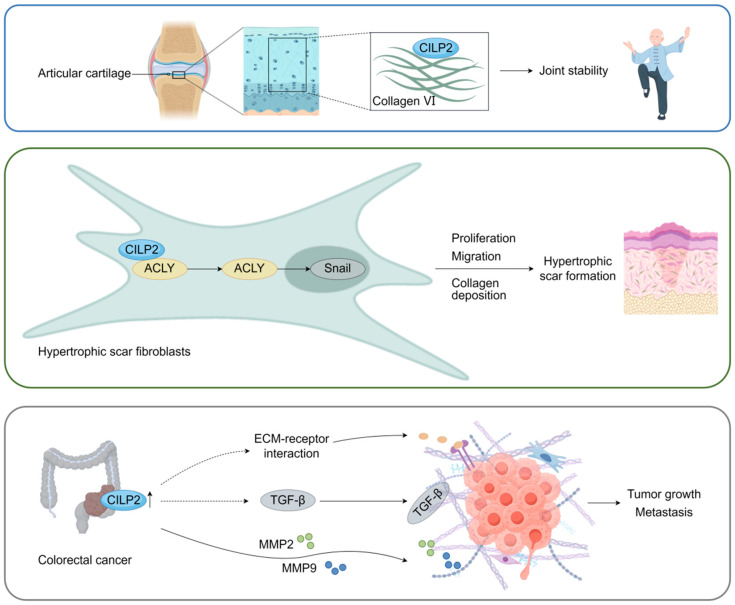
The regulatory roles of CILP2 in ECM organization and remodeling. Top: CILP2 colocalizes with collagen VI in articular cartilage to maintain joint stability. Center: CILP2 interacts with ACLY to stabilize ACLY protein in hypertrophic scar fibroblasts (HSFs). Subsequently, ACLY enhances the acetylation-dependent stabilization of Snail, which promotes the proliferation, migration, and collagen deposition of HSFs, leading to hypertrophic scar formation. Bottom: CILP2 is overexpressed in CRC tissues. CILP2 is associated with ECM-receptor interaction and TGF-β signaling pathways and mediates the production of MMP2 and MMP9, which are critical for tumor growth and metastasis.

In summary, CILP2 serves as a versatile regulator of ECM organization, contributing to physiological structure in cartilage while promoting pathological remodeling in fibrosis and cancer (Figure 3). Its ability to modulate both structural integrity and signaling pathways underscores its potential as a therapeutic target across diverse ECM-driven diseases.

### 3.2. Metabolic Reprogramming

CILP2 is a key regulator of systemic metabolism, influencing glucose and lipid homeostasis across multiple tissues. Elevated serum levels of CILP2 are observed in T2DM, coronary heart disease (CHD) patients, and obese individuals, suggesting its role in metabolic dysregulation [8,9,18]. Mechanistically, in the liver, CILP2 promoted gluconeogenesis through transcriptional activation of phosphoenolpyruvate carboxy kinase (PEPCK), a key gluconeogenic enzyme, while suppressing the phosphorylation of insulin receptor (InsR) and Akt kinase, thereby inducing insulin resistance in T2DM [8]. In high-fat diet (HFD)-fed mice, overexpression of CILP2 recapitulated key metabolic disturbances, including hyperglycemia, dyslipidemia, and systemic insulin resistance [8].

In addition, CILP2 contributes to cardiovascular metabolic pathology by driving atherosclerosis. It was upregulated in the aorta and serum samples of apolipoprotein E knockout (ApoE KO) mice which are widely used for studying atherosclerosis and hypercholesterolemia [9]. It also promoted lipid uptake and foam cell formation in oxLDL-stimulated macrophages via the activation of the peroxisome proliferator-activated receptor-γ (PPAR-γ)/CD36 signaling pathway, linking CILP2 directly to lipid accumulation and plaque development [9].

Sarcopenia is a progressive and generalized skeletal muscle disorder characterized by the accelerated loss of skeletal muscle mass, strength, and function [35]. Sarcopenia is not only related to aging but also considered as both a cause and a consequence of diabetes [36,37]. In the skeletal muscle of aging mice and sarcopenia patients, CILP2 expression was significantly elevated [23]. Mechanistically, Wnt3a was identified as a binding protein of CILP2 via RNA sequencing and co-immunoprecipitation assays. CILP2 interacted with Wnt3a in myoblast cells to inhibit the canonical Wnt signaling and block the nuclear translocation of β-catenin, which impaired mitochondrial biogenesis, glucose metabolism, insulin sensitivity, and myogenesis, leading to muscular atrophy [23]. Notably, silencing CILP2 reversed these defects by improving glucose metabolism, mitochondrial oxidation, and muscle functions in the skeletal muscle of senescence-accelerated mouse P8 (SAMP8) aging mice.

Beyond classical metabolic tissues, CILP2 also participates in ovarian cancer-associated metabolic reprogramming. Based on gene expression profiling, CILP2 was identified as a component of an eight-gene signature associated with the most aggressive molecular subtype of ovarian cancer, characterized by poor prognosis, heightened infiltration of immune cells, elevated carbohydrate metabolism, and enriched oncogenic pathways, including PI3K-Akt and ECM-receptor signaling [28]. CILP2 was upregulated in ovarian cancer and characterized as a risk factor. The eight-gene risk model exhibited strong predictive power in the training set (AUC = 0.83); however, its prognostic accuracy remained moderate in test (AUC = 0.67) and validation (AUC = 0.63) sets [28]. Although further refinement of the model is warranted to enhance its clinical applicability, the inclusion of CILP2 underscores its relevance in tumor metabolic reprogramming and suggests its potential utility in the molecular stratification of ovarian cancer.

**Figure 4:**
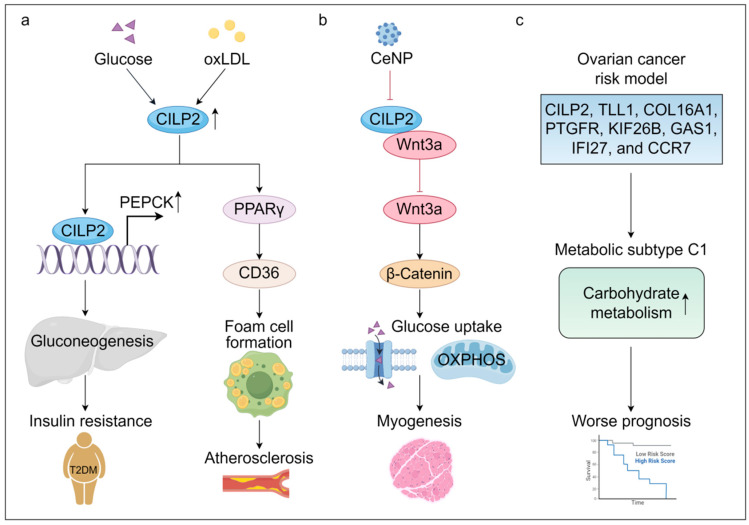
The role and molecular mechanisms of CILP2 in the regulation of metabolic disorders. (**a**) CILP2 is upregulated by metabolic stressors, including high glucose and oxLDL. It drives gluconeogenesis in the liver by transcriptionally activating PEPCK, contributing to insulin resistance in T2DM patients. It also facilitates foam cell formation via PPARγ/CD36 signaling to promote atherosclerosis. (**b**) CILP2 can be suppressed by the antioxidant nanoparticle CeNP. In skeletal muscle, CILP2 binds to Wnt3a and then inhibits canonical Wnt signaling, which impairs glucose uptake and mitochondrial respiration, leading to muscular atrophy. (**c**) CILP2 is a component of an eight-gene signature associated with a unique metabolic subtype of ovarian cancer, which is characterized by elevated carbohydrate metabolism and worse prognosis.

In summary, CILP2 acts as a multi-tissue metabolic modulator, driving insulin resistance, dyslipidemia, muscle atrophy, and tumor metabolic adaptation (Figure 4). Its broad influence across metabolic pathways positions CILP2 as a central node in the pathogenesis of metabolic diseases and a promising target for therapeutic intervention in metabolic disorders.

### 3.3. Supporting Neurodevelopment

As a secreted protein, CILP2 is recognized as a critical regulator in facilitating the communication between the vascular and nervous systems for maintaining neurodevelopment [25]. CILP2 can be secreted by brain endothelial cells (ECs). Silencing CILP2 in brain ECs diverted neural progenitor cells (NPCs) from differentiation to proliferation during early embryonic development, resulting in autistic behaviors in mice [25]. Mechanistically, EC-derived CILP2 interacted with Wnt3a in NPCs and acted as a negative regulator of the canonical Wnt signaling pathway to support normal neurogenesis. The expression and secretion levels of CILP2 were regulated by RNF20, an E3 ubiquitin ligase, required for DNA damage response. Overexpression of CILP2 could partially rescue the endothelial abnormalities and neurogenesis defects caused by RNF20 deficiency in mouse models. However, how RNF20 or DNA damage affects CILP2 expression remains unclear.

### 3.4. EMT Modulation

CILP2 has emerged as a critical driver of tumor progression through its potent regulation of EMT, a key process underlying cancer invasion and metastasis. This functional role is prominently demonstrated in PDAC and CRC.

Pancreatic cancer is the third leading cause of cancer-related deaths [38]. PDAC is the primary type of pancreatic cancer. CILP2 is markedly overexpressed in PDAC and correlated with poor outcomes [7]. Functionally, CILP2 activates the Akt signaling pathway to induce EMT, thereby enhancing tumor cell proliferation, migration, and invasion [7]. In vivo studies showed that CILP2 promoted tumor growth and liver metastasis in orthotopic and metastatic PDAC models.

Similarly, in colorectal cancer, CILP2 was strongly implicated in the development of peritoneal metastases in CRC (PMCRC), which portended the worst prognosis among all metastatic sites [27]. Transcriptomic profiling identified CILP2 as one of the most differentially expressed genes in the primary tumor tissues of CRC patients with peritoneal spread [14]. Mechanistically, CILP2 facilitated metastasis by upregulating EMT markers and enhancing the expression of MMP2 and MMP9, which collectively promoted invasion and peritoneal seeding. Silencing CILP2 effectively suppressed tumor cell growth and migration both in vivo and in vitro.

Importantly, while these findings firmly establish CILP2 as an EMT regulator in PDAC and CRC, several mechanistic questions remain open. The upstream pathways driving CILP2 overexpression in metastatic contexts are not fully elucidated, and the signaling molecules directly regulated by CILP2 require further investigation.

### 3.5. TME Remodeling

Beyond its direct role in driving EMT, CILP2 also profoundly shapes the tumor microenvironment (TME) in PDAC and CRC. PDAC is characterized as an immunologically “cold” tumor, marked by limited infiltration and activation of effector T cells. The majority of PDACs exhibit resistance to immune checkpoint inhibitors (ICIs), largely attributable to the intricate signaling networks within the tumor microenvironment. Immune cell infiltration analyses in PDAC by using the ESTIMATE algorithm showed that high expression of CILP2 was positively correlated with immunosuppressive genes and the infiltration of CAFs and tumor-associated macrophages (TAMs) [7]. This association functionally translated into therapeutic relevance: combining CILP2 silencing with BMS202, a small-molecule PD-1/PD-L1 inhibitor, enhanced tumor regression compared to monotherapy, suggesting CILP2 as a promising target for combination immunotherapy strategies aimed at “heating up” immunologically inert tumors.

In addition, CILP2 is implicated in modulating the immune landscape in CRC. Tumor-infiltrating immune cells (TIICs) are a heterogeneous population of immune cells, such as regulatory T (Treg) cells and M2-type macrophages, that have migrated from the bloodstream into a tumor mass, residing within both the tumor parenchyma and the surrounding stroma [39]. TIICs actively orchestrate the pathogenesis of CRC [40]. High macrophage infiltration and a high ratio of macrophage M2 to macrophage M1 density were associated with poor prognosis in CRC patients [26,41]. CILP2 was included in a macrophage-related prognostic gene signature, with a hazard ratio of 1.482 for predicting CRC outcome, highlighting its clinical relevance [26]. Further immune profiling revealed that CILP2 expression positively correlated with immunosuppressive cell populations, including Tregs, while negatively associating with antitumor immune subsets, including CD4+ T, follicular helper T, and Th17 cells. Additionally, CILP2 was correlated with the expression of immune checkpoint proteins such as PD-1, CD4, and FOXP3, reinforcing its role in fostering an immunosuppressive TME [15].

**Figure 5:**
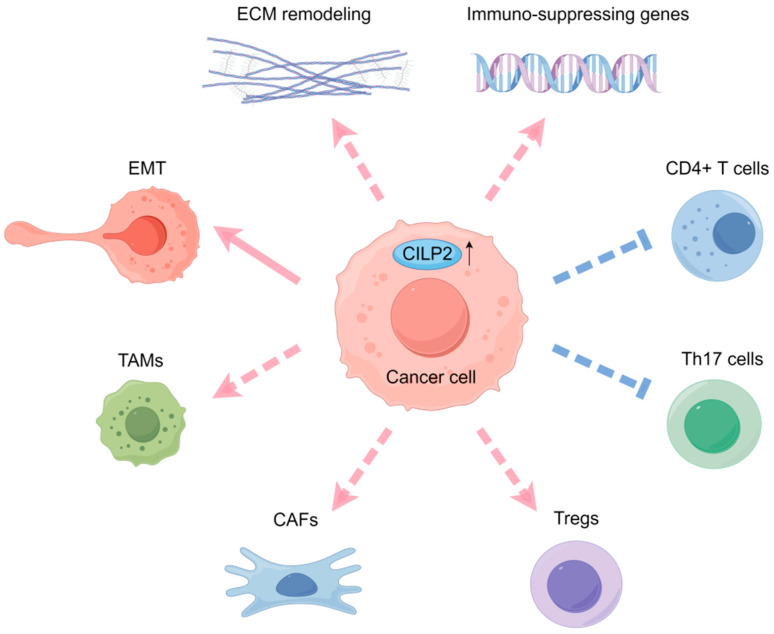
The potential role of CILP2 in the tumor microenvironment. CILP2 promotes metastasis in PDAC and CRC via the EMT process. It is also positively associated with signaling pathways involved in ECM remodeling. High expression of CILP2 was positively correlated with immunosuppressive genes and the infiltration of TAMs, CAFs, Tregs in PDAC and CRC. Conversely, CILP2 is negatively associated with antitumor immune subsets, including CD4+ T and Th17 cells.

Collectively, these findings position CILP2 as a multifaceted regulator of the tumor immune microenvironment (Figure 5). It not only promotes EMT but also facilitates immune evasion by recruiting and modulating immunosuppressive cells. This dual function underscores CILP2’s potential as a therapeutic target, particularly in combination with immune checkpoint inhibitors, to overcome therapeutic resistance and enhance antitumor immunity in cancers such as PDAC and CRC. Future studies should focus on elucidating the precise molecular interactions of CILP2 within the TME to inform targeted therapeutic strategies.

## 4. The Regulation of CILP2

The expression and secretion of CILP2 are dynamically regulated by a variety of physiological and pathological stimuli, reflecting its role as a responsive element within the cellular microenvironment. Current evidence indicates that CILP2 can be upregulated by metabolic and stress-related signals. Metabolically, human serum CILP2 levels are rapidly increased from 115.7 ± 34.3 to 148.7 ± 74.9 ng/L at 2 h after oral glucose uptake, and high glucose conditions directly stimulate the mRNA expression and protein secretion of CILP2 in HepG2 hepatoma cells [8]. In addition, pro-atherogenic lipids, particularly oxLDL, induced mRNA expression and protein secretion of CILP2 in a time- and dose-dependent manner [9].

4-Hydroperoxy cyclophosphamide (4-HC) triggers cellular senescence by inducing DNA damage and a subsequent surge in ROS, serving as a key in vitro model for studying muscle aging and screening anti-sarcopenia therapies. CILP2 expression was upregulated in 4-HC-treated mouse myoblasts, which can be effectively reversed by antioxidant interventions [24]. In addition to oxidative stress, CILP2 was also responsive to DNA damage signals. RNF20 is an E3 ubiquitin ligase that catalyzes the monoubiquitination of histone H2B, which is crucial for regulating gene transcription and facilitating DNA damage repair. The expression and secretion of CILP2 were regulated by RNF20 in brain ECs through an indirect mechanism, as evidenced by the absence of RNF20 binding to the CILP2 promoter in chromatin immunoprecipitation (ChIP) assays [25]. These studies indicate that CILP2 could act as a stress-responsive gene associated with aging, cellular senescence, and neurodevelopment.

Given the high degree of amino acid conservation between CILP1 and CILP2, the upstream regulatory mechanisms of CILP1 may provide critical insights for understanding the regulation of CILP2. CILP1 expression is potently upregulated by TGF-β and bone morphogenetic protein 2 (BMP-2) via the SMAD signaling pathway but is suppressed by IGF-1 [3]. Conversely, CILP1 protein can bind to and antagonize both TGF-β1 and IGF-1, thus establishing negative feedback loops [42,43]. However, as the putative aldehyde dehydrogenase and ATP-binding motifs in the N-terminal domain of CILP1 are not shared in CILP2, CILP2 lacks the specific IGF-1 antagonism [16,17].

Based on current evidence, CILP2 expression is significantly induced by metabolic signals and cellular stress, yet its core upstream regulatory mechanisms remain poorly defined. Key unresolved questions include the following: What are the direct transcriptional regulators driving CILP2 expression? Does CILP2 share regulatory pathways with CILP1, such as TGF-β/BMP-2-SMAD signaling? Finally, does secreted CILP2 merely serve as a stress biomarker, or does it actively modulate the cellular microenvironment through specific receptors or ECM components? Future research must prioritize identifying the direct transcriptional machinery controlling CILP2 and the feedback regulation between CILP2 and the ECM receptor signaling pathways, which will be crucial for understanding the integrated role of CILP2 in tissue homeostasis and disease progression.

## 5. Clinical Significance and Challenges

**Figure 6:**
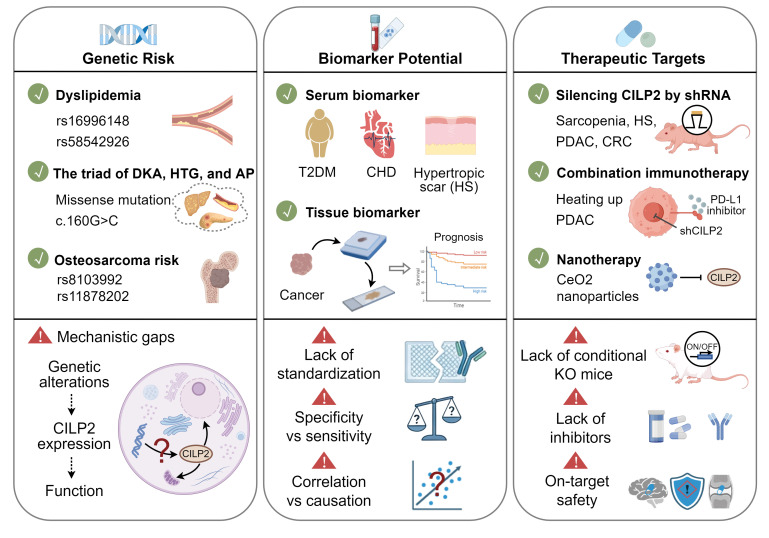
The clinical translatability and challenges of CILP2. Genetic risk (**left**): The CILP2 locus is linked to dyslipidemia (e.g., rs16996148, rs58542926) and osteosarcoma risk (e.g., rs8103992, rs11878202), while a specific missense mutation (c.160G > C) is associated with severe metabolic syndromes. Key mechanistic gaps remain in understanding how these variants alter CILP2 function. Biomarker potential (**center**): Serum CILP2 levels are elevated in patients with T2DM, CHD, and HS, while tissue overexpression of CILP2 predicts poor prognosis in cancer. Clinical application is hindered by lack of assay standardization, unclear diagnostic thresholds, and unproven causality. Therapeutic targets (**right**): CILP2 silencing shows efficacy across diverse preclinical models and can synergize with immunotherapy in PDAC. However, development is challenged by the absence of specific pharmacological tools (e.g., inhibitors, antibodies) and potential on-target safety concerns.

Accumulating evidence establishes CILP2 not only as a key pathogenic driver but also as a molecule of high clinical translatability, with significant implications for diagnosis, prognosis, and therapeutic targeting. However, this promise is accompanied by distinct challenges in each domain, which must be overcome for successful clinical translation (Figure 6).

### 5.1. Genetic Variations and Disease Risk

Dyslipidemia is a major risk factor for cardiovascular and cerebrovascular diseases, caused by multiple environmental and genetic factors [44]. GWAS has firmly established the Neurocan-cartilage intermediate layer protein 2 (NCAN-CILP2) locus, spanning 300 kb on chromosome 19 and forming a tight linkage disequilibrium block, as a genetic hotspot for lipid disorders in individuals of European and East Asian descent [11,19,20,45]. A missense single-nucleotide polymorphism (SNP) of the transmembrane 6 superfamily member 2 (TM6SF2) gene, rs58542926, in the NCAN-CILP2 region, was associated with plasma lipid levels in multiple East Asian ethnic groups and with non-alcoholic fatty liver disease (NAFLD) in Japanese individuals [12]. The other SNP, rs16996148, in the NCAN/CILP2/PBX4 region, located on chromosome 19p13, has also been shown to have significant associations with lipid levels in Europeans, Malays, and Chinese populations [13,19,20,21]. Furthermore, a sex-specific association between the rs16996148 SNP and serum lipid levels was observed in the Mulao and Han ethnic groups within Chinese populations, as the T allele carriers had higher serum lipid levels than the subjects with the GG genotype in males but not in females [13]. Beyond common SNPs, a heterozygous missense mutation, c.160G > C (p.Glu54Gln), in CILP2 was linked to the triad of diabetic ketoacidosis, hypertriglyceridemia, and acute pancreatitis, a severe metabolic syndrome [22].

Osteosarcoma, the most common primary malignant bone tumor, typically occurs in adolescents and frequently arises in rapidly growing bone regions [46]. Taller stature is identified as a risk factor for osteosarcoma [47]. Notably, the height-associated SNPs rs8103992 and rs11878202, which are expression quantitative trait loci (eQTLs) for the CILP2 gene, are significantly associated with osteosarcoma risk [29]. Moreover, the risk allele (A) of rs8103992 is located within a putative osteoblast enhancer region and predicted to preserve binding sites for transcription factors, including forkhead box A2 (FOXA2), hepatocyte nuclear factor 4 alpha (HNF4A), and the EWS RNA binding protein 1 (EWSR1) fusion protein found in Ewing sarcoma, suggesting a potential mechanism by which it may influence both bone growth and oncogenesis.

These studies underscore the genomic locus of CILP2 as a key genetic nexus influencing metabolic disorders and osteosarcoma risk. Looking ahead, major translational challenges lie in deciphering the precise molecular mechanisms and functions of these genetic variations that drive such diverse pathological outcomes.

### 5.2. Biomarker

CILP2 emerges as a promising serum biomarker for non-invasive diagnosis and monitoring of diseases. Serum CILP-2 levels were significantly elevated in patients with impaired glucose tolerance (IGT), T2DM, CHD, and hypertrophic scars [8,9,10]. In CHD, serum CILP2 levels were positively correlated with Gensini scores, a quantitative assessment tool used to evaluate the severity of coronary artery stenosis based on angiographic findings [9]. In T2DM patients, CILP2 levels were positively correlated with waist-to-hip ratio (WHR), fasting blood glucose (FBG), 2-h blood glucose after glucose overload (2h-BG), HbA1c, fasting insulin, 2-h plasma insulin after glucose overload, triglyceride (TG), and insulin resistance but negatively correlated with high-density lipoprotein cholesterol (HDL-C) levels [8]. Moreover, serum CILP2 levels were rapidly increased after oral glucose uptake and were significantly decreased in T2DM patients after 12 weeks of anti-diabetic treatment with Exenatide, suggesting that serum CILP2 could be a potential biomarker for the therapeutic monitoring of T2DM.

Recently, CILP2 has also been recognized as a potential prognostic biomarker in cancer [7]. Bioinformatic analysis based on The Cancer Genome Atlas (TCGA) database showed that CILP2 was overexpressed in most cancer tissues and positively correlated with poor outcomes of most cancer types, such as kidney cancer, low-grade glioma, hepatocellular carcinoma, skin cutaneous melanoma, and thyroid carcinoma [7]. Notably, the prognostic predictive role of tissue CILP2 was confirmed in several independent cohorts of CRC patients [7,14,34].

The above evidence positions serum CILP2 as a promising tool for diagnosis and treatment monitoring in metabolic diseases and indicates that tissue CILP2 could be a potential prognostic biomarker in cancer. However, the clinical translation of CILP2 as a biomarker faces several challenges. Firstly, lack of standardization in detection methods remains a major barrier. Although CILP2 ELISA kits and immunohistochemical antibodies are commercially available, they have not been validated for clinical use, limiting consistent measurement and interpretation across laboratories. Secondly, the establishment of specificity versus sensitivity is complicated by the absence of disease-specific diagnostic thresholds, while confounding variables—such as obesity, aging, and metabolic comorbidities—may influence CILP2 levels and have not been adequately accounted for in existing studies. Finally, while CILP2 shows strong correlative associations with disease outcomes, causality and clinical utility remain uncertain, particularly in oncology, where most evidence derives from bioinformatic analyses and retrospective cohorts. Therefore, prospective validation in well-designed clinical studies is essential to define actionable cut-offs and ultimately enable its integration into routine diagnostic and prognostic workflows.

### 5.3. Therapeutic Target

The diverse and potent pathophysiological roles of CILP2 establish it as an attractive and novel therapeutic target across multiple diseases. Converging in vivo evidence demonstrates that silencing CILP2 via genetic approaches yields significant therapeutic benefits. In sarcopenia models, CILP2 inhibition reversed glucose intolerance and mitochondrial dysfunction in aged skeletal muscle, leading to the restoration of muscle function [23]. In hypertrophic scar models, silencing CILP2 attenuated dermal fibrosis and collagen deposition in dermal fibrotic lesions [10]. In cancer biology, CILP2 functioned as a driver of tumor progression [7]. Silencing CILP2 inhibited primary tumor growth and metastasis in PDAC and CRC models [7,14]. Notably, in immunologically “cold” tumors like PDAC, where resistance to immunotherapy is common, silencing CILP2 synergized with the PD-1/PD-L1 inhibitor enhanced antitumor efficacy, likely by counteracting its role in fostering an immunosuppressive microenvironment rich in CAFs and TAMs [7]. Beyond direct targeting, indirect targeting via antioxidative agents such as synthesized cerium oxide (CeO2) nanoparticles (CeNPs) reduced CILP2 expression and showed anti-aging effects in mouse muscle, confirming its druggability [24].

Despite compelling preclinical evidence, the path toward clinical translation of CILP2-targeted therapies faces several interconnected hurdles. First, the lack of conditional knockout mouse models limits our understanding of CILP2’s specific functions across different tissues. Second, there are currently no small-molecule inhibitors or neutralizing antibodies available for CILP2, which are essential for both mechanistic studies and therapeutic development. Finally, safety concerns exist because CILP2 has important physiological roles in joint stability and neurodevelopment. Systemic inhibition of CILP2 might cause unintended side effects in these systems, underscoring the need for tissue-specific targeting and careful definition of the therapeutic window. Addressing these gaps will be essential for advancing CILP2 from a promising target to a clinically viable therapy.

## 6. Conclusions

CILP2 emerges as a pivotal molecular node across diverse pathologies due to its dual role as both a structural ECM modulator and a pleiotropic signaling regulator. Its broad expression and stress-responsive upregulation—triggered by metabolic and oxidative cues—enable it to act as a molecular integrator across diverse disease contexts. Structurally, domains like TSP-1 of CILP2 might facilitate its interactions with ECM components such as collagen VI, ECM receptors, and signaling molecules (e.g., Wnt3a and ACLY). These interactions may enable CILP2 to participate in different biological processes—from ECM assembly to signal modulation—depending on the cellular context and binding partners. Functionally, CILP2 regulates ECM remodeling to maintain joint stability and promotes hypertrophic scar formation. It is also associated with the TGF-β signaling pathway, ECM-receptor interaction, and ECM degradation in CRC. In metabolic disorders, CILP2 regulates PEPCK, PPARγ/CD36, and canonical Wnt signaling pathways to modulate glucose, lipid, and mitochondrial metabolism, leading to insulin resistance, atherosclerosis, and sarcopenia, respectively. In addition, secreted by brain endothelial cells, CILP2 supports neurovascular crosstalk and neurogenesis by blocking Wnt signaling in neural progenitor cells. In cancer, CILP2 functions as an oncoprotein and activates EMT in PDAC and CRC. It may also foster an immunosuppressive TME by correlating with increased infiltration of CAFs, TAMs, and Tregs and is associated with resistance to immunotherapy in PDAC. Furthermore, genetic variations of CILP2 link it to metabolic disorders and osteosarcoma risk, while its consistent dysregulation in patient serum and tissues underscores its translational potential as a diagnostic and prognostic biomarker. Collectively, the structural adaptability, signaling plasticity, and stress-responsive expression of CILP2 elucidate how a single molecule can orchestrate diverse pathological processes—from metabolic dysfunction and fibrosis to neurodevelopmental regulation and oncogenesis. These insights not only clarify its pleiotropic mechanisms but also highlight CILP2 as a promising biomarker and a compelling target for integrated therapeutic strategies across multiple disease contexts.

## 7. Future Perspectives

Future research on CILP2 is poised to delve into the mechanistic core of its pleiotropy, with a focus on its structural duality and context-dependent regulation. A central and unresolved question is how its distinct protein domains—particularly the conserved N-terminal TSP-1 and Ig C-2 domains—orchestrate its diverse functions. Protein-protein interaction studies and molecular docking analyses are critically needed to elucidate the precise binding interfaces between CILP2 and its known partners, such as Collagen VI, Wnt3a, and ACLY, as well as to identify novel interacting molecules. Moreover, as an ECM component, identifying the specific cellular receptors for CILP2 is a crucial research priority. As a secreted glycoprotein, CILP2 could act in a paracrine or autocrine manner, thereby regulating intra- and extracellular functions. For example, CILP2 secreted by brain endothelial cells binds to Wnt3a in neural precursor cells to regulate neurogenesis, while in muscle, cellular CILP2 interacts with Wnt3a to suppress myogenesis [23,25]. Elucidating its receptor interactions and revealing potential autocrine or paracrine feedback loops is essential to understanding the molecular basis of CILP2 in ECM modification and TME remodeling.

Simultaneously, research must progress to systematically mapping the regulatory network of CILP2. While CILP2 expression is known to be induced by metabolic stimuli and cellular stress, the direct transcriptional regulators and signaling cascades governing its basal and inducible expression remain largely undefined. A critical line of investigation is to elucidate whether CILP2 is regulated by the TGF-β/BMP-SMAD pathway, akin to CILP1, and how this potential axis integrates with stress-activated transcription factors. Furthermore, while CILP2 has been implicated in activating key signaling pathways such as PPARγ/CD36 and Akt/EMT, the precise mechanism of its involvement—whether through direct interaction with signaling molecules or indirect modulation via upstream regulators or the ECM—remains to be elucidated.

Beyond mechanistic inquiry, the clinical translation of CILP2 requires addressing key gaps in its application as a biomarker and a therapeutic target. For its biomarker potential, future efforts must focus on standardizing detection assays and defining disease-specific diagnostic thresholds through large-scale, prospective validation studies. It is crucial to determine whether CILP2 elevation is a causal driver or a secondary consequence of disease, while accounting for confounding metabolic and inflammatory variables. Therapeutically, the development of pharmacologic CILP2 inhibitors—such as small molecules or neutralizing antibodies—is essential. Given its physiological roles, strategies for tissue-selective targeting must be prioritized to mitigate potential side effects. In oncology, combining CILP2 blockade with immunotherapy represents a promising strategy, particularly for immunologically “cold” tumors. Advancing these approaches into clinical trials will be critical to evaluate the safety and efficacy of CILP2-targeted interventions across different disease contexts.

## Figures and Tables

**Table 1:** The roles and mechanisms of CILP2 in diseases.

Disease	Dysregulation	Function	Mechanism	Research Objects	Reference
Osteoarthritis	Reduced in damaged cartilage	Maintains the joint stability of mice	Interacts with Collagen VI	Mice experimental osteoarthritis	[6]
T2DM	Increased in serum	Promotes gluconeogenesis and insulin resistance	Upregulates PEPCK and suppress InsR and Akt kinase	Chinese population, HFD-fed mice, HepG2 cells, mouse primary hepatocytes	[8]
Obese	Increased in serum	Lipid metabolism and insulin resistance	NA	Chinese population	[18]
CHD	Increased in serum	Lipid uptake and foam cell formation	Activates PPARγ/CD36 signaling axis	Chinese population, ApoE KO mice, THP-1 cells	[9]
Dyslipidemia	rs58542926 in NCAN-CILP2 region	Associated with plasma lipid levels	NA	East Asian ethnic groups	[12]
Dyslipidemia	rs16996148 in NCAN/CILP2/PBX4 region	Associated with plasma lipid levels	NA	Europeans, Malays, and Chinese populations	[13,19,20,21]
The triad of DKA, hypertriglyceridemia, and AP	c.160G > C (p.Glu54Gln) in CILP2	Associated with plasma lipid levels	NA	A Chinese patient	[22]
Sarcopenia	Increased in serum in skeletal muscle	Impairs glucose metabolism and mitochondrial function, leading to muscular atrophy	Suppresses the Wnt/β-catenin signaling axis	Chinese population, SAMP8 mice, C2C12 cells	[23]
Sarcopenia	Increased in serum in ROS-treated aging muscle tissues and satellite cells	CeNP, a potential inhibitor of sarcopenia, suppresses CILP2 expression	Upregulates SERPINE1 and phos-pho-p21 related to the inflammatory response	CeNP-treated C57/BL6J mice (aged 52–54 weeks), C2C12 cells	[24]
Hypertrophic scar (HS)	Increased in serum and tissues	Activates HSFs and promotes collagen deposition	Interacts with ACLY to stabilize Snail	Chinese patients with HS, mouse and rabbit HS models, HSFs	[10]
Neurodevelopment	Decreased in RNF20-deleted brain ECs	Maintains neurovascular interaction and promote neurogenesis	Interacts with Wnt3a and suppresses the canonical Wnt signaling pathway	Endothelial RNF20 conditional knockout mice, primary mouse brain ECs and NPCs	[25]
PDAC	Increased in tissues and cell lines	Promotes cell proliferation, migration, and invasion; Silencing CILP2 sensitizes PDAC to ICIs	Activates Akt/EMT signaling; Correlated with CAFs/TAMs infiltration and expression of immuno-suppressing genes	Chinese PDAC patients, BALB/c ortho-topic and metastatic PDAC mice models, C57BL/6 orthotopic PDAC mice model, mouse and human PDAC cell lines	[7]
CRC	Increased in tissues	Associated with ECM-related function and Treg infiltration	Associated with ECM–receptor interaction, the TGF-β signaling pathway, and the expression of PD-1, CD4, and FOXP3	Chinese CRC patients, human CRC cell lines	[15]
CRC	Independent risk factor to predict prognosis	Associated with macrophage infiltration	NA	TCGA CRC dataset	[26]
PMCRC	Increased in tissues	Promotes tumor growth and metastasis	Regulates MMP2, MMP9, and EMT markers	Korean PMCRC patients, BALB/c PM metastatic CRC mice model, human CRC cell lines	[27]
OV	Increased in tissues; risk factor to predict prognosis	Associated with a unique metabolic phenotype	NA	TCGA OV dataset	[28]
Osteosarcoma	rs8103992 and rs11878202, eQTL for CILP2	Associated with osteosarcoma risk	NA	California OS patients, dbGaP study accession phs000734.v1.p1 and phs000381.v1.p1 datasets	[29]

## Data Availability

No new data were created or analyzed in this study. Data sharing is not applicable to this article.

## Abbreviations

The following abbreviations are used in this manuscript:

2h-BG	2-h blood glucose after glucose overload
4-HC	4-Hydroperoxy cyclophosphamide
ACLY	ATP citrate lyase
ApoE KO	Apolipoprotein E knockout
ASO	Antisense oligonucleotide
BMP-2	Bone morphogenetic protein 2
CAFs	Cancer-associated fibroblasts
CeNPs	Cerium oxide (CeO2) nanoparticles (CeNPs)
CHD	Coronary heart disease
ChIP	Chromatin immunoprecipitation
CILP2	Cartilage intermediate layer protein 2
CRC	Colorectal cancer
ECM	Extracellular matrix (ECM)
ECs	Endothelial cells
EMT	Epithelial-mesenchymal transition
eQTL	Expression quantitative trait locus
FBG	Fasting blood glucose
GAG	Glycosaminoglycan
GWAS	Genome-wide association studies
HDL-C	High-density lipoprotein cholesterol
HFD	High-fat diet
HS	Hypertrophic scar
HSFs	Hypertrophic scar fibroblasts
ICIs	Immune checkpoint inhibitors
Ig	Immunoglobulin
IGF-1	Insulin-like growth factor-1
IGT	Impaired glucose tolerance
InsR	Insulin receptor
LDL	Low-density lipoprotein cholesterol
MMP2	Matrix metalloproteinase 2
NAFLD	Non-alcoholic fatty liver disease
NCAN-CILP2	Neurocan-cartilage intermediate layer protein 2
NPCs	Neural progenitor cells
NPPs	Nucleotide pyrophosphatase phosphodiesterase
NS	Normal skin
OV	Ovarian cancer
oxLDL	Oxidatively modified LDL
PDAC	Pancreatic ductal adenocarcinoma
PEPCK	Phosphoenolpyruvate carboxy kinase
PMCRC	Peritoneal metastases in CRC
PPARγ	Peroxisome proliferator-activated receptor-γ
ROS	Reactive oxygen species
RT-qPCR	Quantitative real-time PCR
Treg	Regulatory T
SAMP8	Senescence-accelerated mouse P8
SNP	Single-nucleotide polymorphism
T2DM	Type 2 diabetes mellitus
TAMs	Tumor-associated macrophages
TC	Total cholesterol
TG	Triglyceride
TGF-β1	Transforming growth factor beta 1
TIMER	Tumor Immune Estimation Resource
TIICs	Tumor-infiltrating immune cells
TM6SF2	Transmembrane 6 superfamily member 2
TME	Tumor microenvironment
TSP	Thrombospondin
WHR	Waist-to-hip ratio
